# Reliability of brain volume measures of accelerated 3D T1-weighted images with deep learning-based reconstruction

**DOI:** 10.1007/s00234-024-03461-5

**Published:** 2024-09-24

**Authors:** Woojin Jung, Geunu Jeong, Sohyun Kim, Inpyeong Hwang, Seung Hong Choi, Young Hun Jeon, Kyu Sung Choi, Ji Ye Lee, Roh-Eul Yoo, Tae Jin Yun, Koung Mi Kang

**Affiliations:** 1 AIRS Medical, 223, Teheran-ro, Gangnam-gu, Seoul, 06142 Republic of Korea; 2 Department of Radiology, Seoul National University Hospital, 101 Daehak-ro, Jongno-gu, Seoul, 03080 Republic of Korea; 3 Department of Radiology, Seoul National University College of Medicine, 103 Daehak- ro, Jongno-gu, Seoul, 03080 Republic of Korea

**Keywords:** Magnetic resonance imaging, Brain, Volumetry, Deep learning

## Abstract

**Purpose:**

The time-intensive nature of acquiring 3D T1-weighted MRI and analyzing brain volumetry limits quantitative evaluation of brain atrophy. We explore the feasibility and reliability of deep learning-based accelerated MRI scans for brain volumetry.

**Methods:**

This retrospective study collected 3D T1-weighted data using 3T from 42 participants for the simulated acceleration dataset and 48 for the validation dataset. The simulated acceleration dataset consists of three sets at different simulated acceleration levels (Simul-Accel) corresponding to level 1 (65% undersampling), 2 (70%), and 3 (75%). These images were then subjected to deep learning-based reconstruction (Simul-Accel-DL). Conventional images (Conv) without acceleration and DL were set as the reference. In the validation dataset, DICOM images were collected from Conv and accelerated scan with DL-based reconstruction (Accel-DL). The image quality of Simul-Accel-DL was evaluated using quantitative error metrics. Volumetric measurements were evaluated using intraclass correlation coefficients (ICCs) and linear regression analysis in both datasets. The volumes were estimated by two software, NeuroQuant and DeepBrain.

**Results:**

Simul-Accel-DL across all acceleration levels revealed comparable or better error metrics than Simul-Accel. In the simulated acceleration dataset, ICCs between Conv and Simul-Accel-DL in all ROIs exceeded 0.90 for volumes and 0.77 for normative percentiles at all acceleration levels. In the validation dataset, ICCs for volumes > 0.96, ICCs for normative percentiles > 0.89, and R^2^ > 0.93 at all ROIs except pallidum demonstrated good agreement in both software.

**Conclusion:**

DL-based reconstruction achieves clinical feasibility of 3D T1 brain volumetric MRI by up to 75% acceleration relative to full-sampled acquisition.

**Supplementary Information:**

The online version contains supplementary material available at 10.1007/s00234-024-03461-5.

## Introduction

Distinct patterns of brain atrophy in specific regions have the potential to distinguish between normal individuals and those with dementia, providing valuable insights into disease progression. Hippocampal and medial temporal atrophy is a key imaging biomarker for Alzheimer’s disease. Magnetic resonance imaging (MRI) offers high-resolution depictions of brain anatomy and allows for detailed visualization of structural changes in the brain, particularly through three-dimensional (3D) T1-weighted images [1]. As subjective visual assessment for diagnosing brain atrophy yields limited consensus and notable interobserver variability [2], various automated algorithms have been employed for brain volumetric analysis in research settings, including FreeSurfer [3, 4], ANTs [5] and FSL [6].

Conducting MRI-based brain volumetry in clinical practice encounters several challenges due to the time-intensive nature of acquiring 3D T1-weighted images and analyzing brain volumetry. Prolonged acquisition time can lead to motion artifacts and patient discomfort, resulting in image quality degradation [7, 8]. Additionally, extended analysis times and complex algorithms further contribute to time-consuming and intricate aspects of clinical implementation. For example, analysis time for brain volumetry has been reported as 2 to 3 h for FreeSurfer (9) and 1 to 2 h for ANTS [9]. However, recent advancements in deep learning (DL)-based reconstruction algorithms have significantly reduced the time required for image acquisition [10–13]. Clinically practical automated brain volumetric software can process data within 10 min or less [14–19].

Accelerating the acquisition of 3D T1-weighted images with DL-based reconstruction and developing automated brain volumetric software will hasten the clinical utilization of volumetric results. Nonetheless, several issues merit attention in this context. Although previous studies have reported that the image quality of accelerated scans with DL-based reconstruction is not inferior to conventional scans [12, 20–22], few studies have addressed assessing brain volume measurement reliability in accelerated scans with DL-based reconstruction [23, 24]. Furthermore, despite the option to adjust the acceleration level, the current body of research lacks an exploration into evaluating the diverse acceleration levels necessary to maintain consistent volumetric results. In addition, there remains a lack of investigation into whether accelerated scans with DL-based reconstruction produce consistent volumetric outcomes compared to those from conventional scans when various software approaches are employed.

This study investigated the reliability and clinical feasibility of automated brain volume measures obtained from accelerated MRI scans with DL-based image reconstruction at various acceleration levels. This included assessing inter-scan reliability between conventional scans and those with DL-based reconstruction using two different clinically available volumetric software.

## Methods and materials

### Participants

This retrospective study was approved by the institutional review board (IRB) at Seoul National University Hospital. Informed consent from the participants was waived due to the retrospective nature of this study. Two datasets – the simulated acceleration and validation datasets - were collected. For the simulated acceleration dataset, 42 consecutive participants who performed to determine the presence of brain metastasis but exhibited no abnormalities in the brain were recruited over eight months. For the validation dataset, 48 consecutive participants who underwent brain MRI for health screening were recruited over eight months.

### Image acquisition

All images were acquired on a 3T MR scanner with a 32-channel array head coil (MAGNETOM Skyra, Siemens Healthineers, Erlangen, Germany). 3D magnetization-prepared rapid gradient-echo (MP-RAGE) k-space data were collected using conventional scanning protocols for the simulated acceleration dataset. Acquisition parameters of the protocol were as follows: TR = 1,600 ~ 1,740 ms, TE = 2.8 ms, TI = 900ms, flip angle = 9°, voxel size = 1 × 1 × 1 mm^3^, phase resolution = 100%, and generalized auto-calibrating partial parallel acquisition (GRAPPA) factor = 2. The scan time ranged between 180 and 214 s.

Participants underwent conventional and accelerated scans for 3D MP-RAGE DICOM data for the validation dataset. The scan parameters for conventional scan data were the same as those for the simulated acceleration dataset, while those of the accelerated scan were modified as follows: TR = 1,600 ms, phase resolution = 60%, and GRAPPA factor = 3. The resulting accelerated scan time was 100 s.

### Image processing

This study utilized two different datasets: the simulated acceleration dataset and the validation dataset. In the simulated acceleration dataset, k-space data were collected to simulate various acceleration levels. Therefore, the variation of the volume measurements across the acceleration level can be measured. On the other hand, the validation dataset consists of two different scans: conventional scan and accelerated scan. The dataset was utilized to evaluate the inter-scan reliability of the proposed framework.

**Fig. 1:**
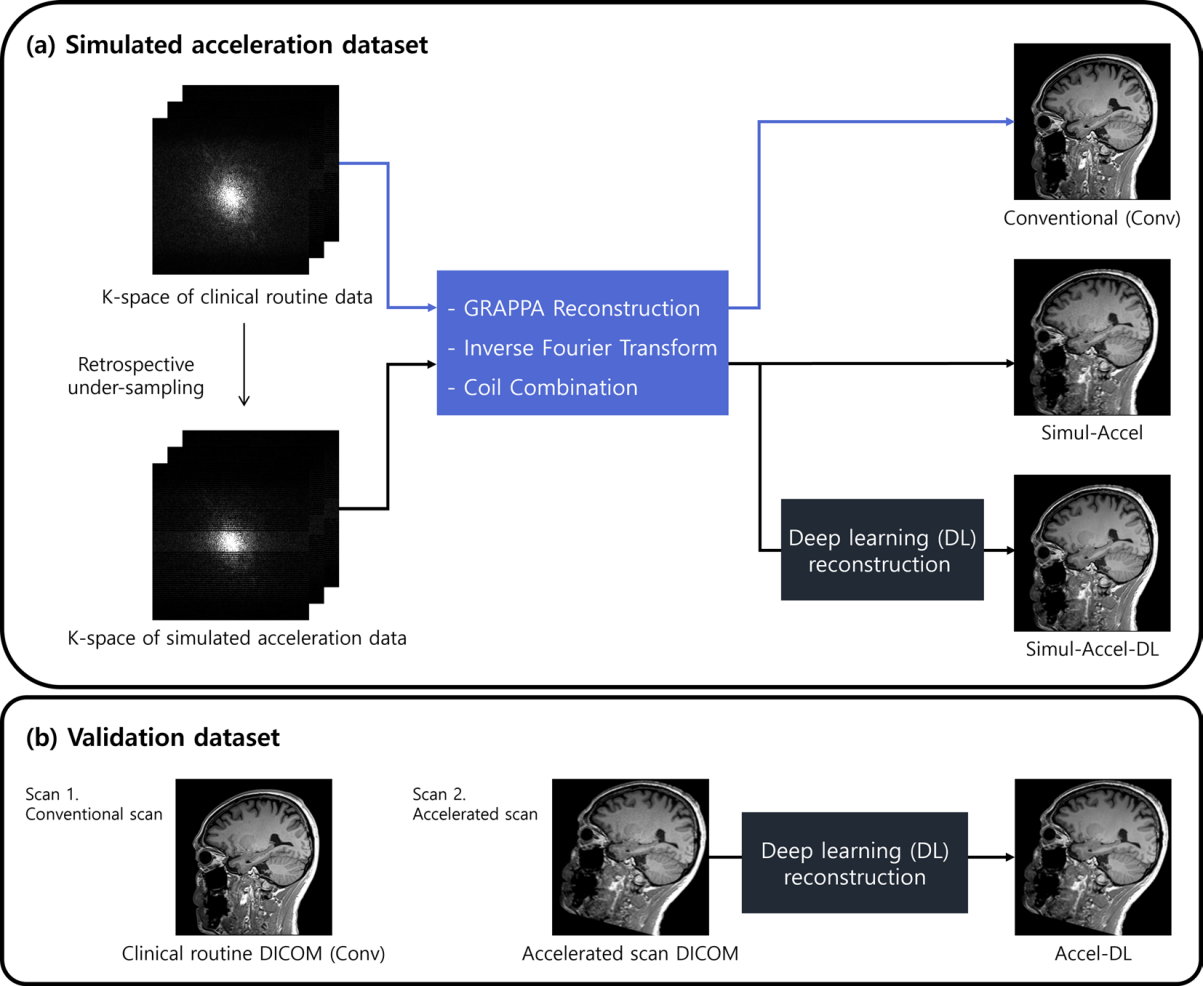
(**a**) Simulated acceleration dataset used k-space data acquired from conventional scans. From the k-space data, three different DICOM images, conventional images (Conv), simulated acceleration images (Simul-Accel), and simulated acceleration images with DL-based reconstruction (Simul-Accel-DL) were generated. (**b**) The validation dataset consisted of Conv and accelerated scans with deep learning-based reconstruction (Accel-DL)

In the simulated acceleration dataset, three images were generated using the acquired k-space data, as shown in Fig. 1a. The first type, conventional images (Conv), was reconstructed from the k-space data by sequentially applying GRAPPA reconstruction, inverse Fourier transform, and channel combination. Second, simulated acceleration images (Simul-Accel) were generated from the retrospectively under-sampled k-space data followed by the same reconstruction process of Conv. Lastly, DL-based reconstruction was applied to Simul-Accel, generating Simul-Accel-DL. DL-based reconstruction was conducted using a clinically available DICOM-based image post-processing software (SwiftMR^®^, v.2.0.0.0., AIRS Medical, Seoul, Republic of Korea). This software could image denoise and enhance resolution in the image domain. The deep neural network architecture utilized in the software was based on a variant of U-net composed of 18 convolutional blocks, four max-pooling layers (pool size = 2 × 2), four upsampling layers (kernel size = 2 × 2), four feature concatenations, and three convolutional layers (kernel size = 1 × 1). The network was trained on pairs of 3D MR images, where one image in each pair had low SNR and low resolution, and the other had high SNR and high resolution. The training data included 3D MR images collected from over 1,000 patients on both 1.5T and 3T MR scanners.

To compare Conv, Simul-Accel, and Simul-Accel-DL across various levels of acceleration, three different under-sampling schemes were simulated by varying phase resolutions from 0.4 to 0.6 with an interval of 0.1. For each level of acceleration, the estimated simulated scan time was estimated as follows: 128 ~ 152 s for a phase resolution of 0.6 (Level 1), 109 ~ 130 s for 0.5 (Level 2), and 91 ~ 109 s for 0.4 (Level 3). Note that levels 1, 2, and 3 were accelerated by 65%, 70%, and 75% relative to full-sampled acquisition, respectively.

In the validation dataset, both conventional (Conv) and accelerated scans were acquired in each participant (Fig. 1b). The scan time of the accelerated scans was set to be equivalent to the Level 3 simulation. DL-based reconstruction in the simulated acceleration dataset was applied to the accelerated scan images (Accel-DL).

### Image analysis

Quantitative analysis for image quality was performed with the simulated acceleration dataset. Quantitative error metrics, structural similarity index (SSIM), and peak signal-to-noise ratio (PSNR) were calculated at Simul-Accel and Simul-Accel-DL by Conv as a reference. SSIM represents the similarity of signal intensity in local regions [25, 26]. Higher SSIM and PSNR indicate better image quality.

For volumetric analysis, clinically available software based on machine learning, NeuroQuant (NeuroQuant^®^, v.3.1., Cortechs.ai, San Diego, USA) and Deepbrain (DeepBrain^®^, v.1.1.1.2, Vuno, Seoul, Korea) were utilized. The software analyses 3D T1-weighted DICOM images as inputs and provides volumetric measurement of brain structures and normative percentiles based on the subject’s age and sex.

### Statistical analysis

For the image quality evaluation, a paired t-test was performed between Simul-Accel and Simul-Accel-DL results to demonstrate statistical significance (*p* < 0.05). Inter-scan reliability of volumes and normative percentiles between Conv and either Simul-Accel-DL or Accel-DL was estimated by intraclass correlation coefficient (ICC) using a 2-way mixed model with consistency type. ICC values were classified on a 4-grade scale: poor (< 0.5), moderate (0.50–0.75), good (0.75–0.90), and excellent (> 0.9) [27]. The simulated acceleration dataset calculated ICC between Conv and Simul-Accel-DL for each brain region of interest (ROI) across acceleration levels. The validation dataset calculated ICC between Conv and Accel-DL in each brain ROI.

To demonstrate the agreement of volume measurement, regression coefficient and R-squared values were estimated through linear regression analysis in each ROI. Additionally, the volumes between Conv and Accel-DL were compared in ROIs representing biomarkers for neurodegenerative disease such as hippocampus and inferior lateral ventricles, using linear regression and Bland-Altman plots. All statistical analyses were performed using SPSS, Version 28.0 (IBM Corp., Armonk, NY, USA).

## Results

### Demographic characteristics

The demographic characteristics of the included participants have been presented in Table 1. The simulated acceleration dataset was acquired from 42 participants with mean age ± standard deviation of 66 ± 11 years. The dataset consisted of 14 female and 28 male data. In the validation dataset, 48 participants data with mean age ± standard deviation of 61 ± 9 years were acquired, where the dataset consisted of 23 female and 25 male data.

**Table 1:** Demographic characteristics

Characteristic	Value
Simulated acceleration dataset	
Sequence	MP-RAGE
No. of participants	42
Age (years)*	66 ± 11
No. of female/male participants	14/28
Validation dataset	
Sequence	MP-RAGE
No. of participants	48
Age (years)*	61 ± 9
No. of female/male participants	23/25

*Note* Ages are presented as means ± standard deviations. MP-RAGE = magnetization-prepared rapid gradient-echo

### Quantitative error metric

**Fig. 2:**
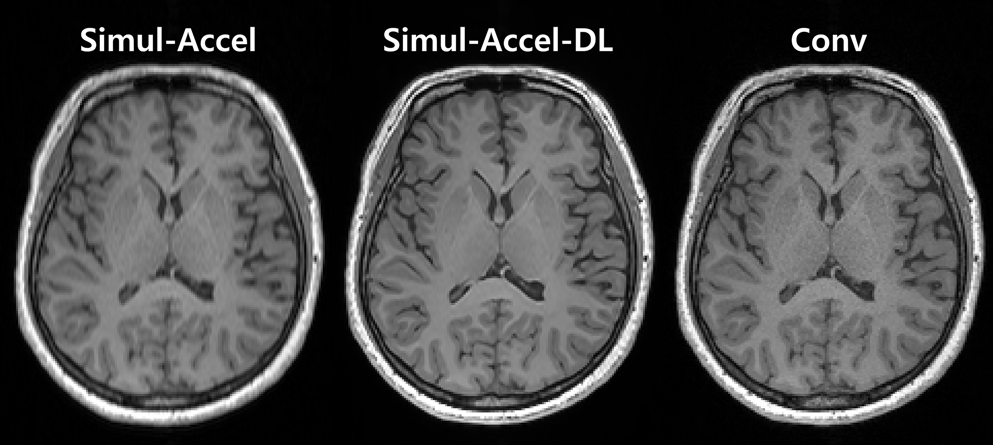
The representative Simul-Accel, Simul-Accel-DL, and Conv images from a 60-year-old woman are shown. Simul-Accel-DL shows better image quality than Simul-Accel, supporting the quantitative error metric results

In the simulated acceleration dataset, overall image quality demonstrated comparable or better metrics in Simul-Accel-DL than in Simul-Accel, with reference to Conv (Table 2). As the acceleration level increased, the difference in image quality between Simul-Accel and Simul-Accel-DL increased. Table 2 presents the result of the quantitative error metric across different acceleration levels between Simul-Accel and Simul-Accel-DL. At the lowest acceleration level, level 1, both SSIM and PSNR were comparable between Simul-Accel and Simul-Accel-DL, with mean SSIMs of 0.98 ± 0.01 in Simul-Accel-DL and 0.98 ± 0.01 in Simul-Accel (*p* = 0.815), and the mean PSNRs of 37.0 ± 2.0 in Simul-Accel-DL and 36.8 ± 3.0 in Simul-Accel (*p* = 0.330). However, at higher acceleration levels 2 and 3, Simul-Accel-DL demonstrated statistically superior to Simul-Accel. The mean of SSIM in Simul-Accel-DL was 0.01 higher at acceleration level 2 (0.97 vs. 0.98) and 0.02 higher at acceleration level 3 (0.95 vs. 0.97), indicating that the Simul-Accel-DL image had a higher structural similarity with conventional scan (*p* < 0.001). The mean of PSNR in Simul-Accel-DL was also higher than in Simul-Accel images at both acceleration levels (*p* < 0.001). Figure 2 shows examples images of Simul-Accel, Simul-Accel-DL and Conv in accelerated level 3, supporting the better error metrics of Simul-Accel-DL in Table 2.

**Table 2:** Comparison of quantitative error metrics between Simul-Accel and Simul-Accel-DL at different acceleration levels

Quantitative error metric	Level 1 (65% reduction)		Level 2 (70% reduction)		Level 3 (75% reduction)	
	Simul-Accel	Simul-Accel-DL	Simul-Accel	Simul-Accel-DL	Simul-Accel	Simul-Accel-DL
Structural similarity index (0–1)	0.98 ± 0.01	0.98 ± 0.01	0.97 ± 0.01	0.98 ± 0.01	0.95 ± 0.01	0.97 ± 0.01
	*P* = 0.815		*P < 0.001		*P < 0.001	
Peak signal-to-noise ratio (dB)	37.0 ± 2.0	36.8 ± 3.0	34.9 ± 1.8	35.7 ± 2.1	32.6 ± 1.6	34.5 ± 1.6
	*P* = 0.330		*P < 0.001		*P < 0.001	

*Note* Mean and standard deviation were calculated across the participants. Acceleration levels 1, 2, and 3 were simulated by 65%, 70%, and 75% undersampling relative to full-sampled acquisition, respectively. Simul-Accel = Images reconstructed from simulated acceleration k-space data and Simul-Accel-DL = Simul-Accel images with deep learning-based reconstruction

* indicate statistically significant differences between Simul-Accel and Simul-Accel-DL (*P* < 0.05)

### Volumetric analysis

**Fig. 3:**
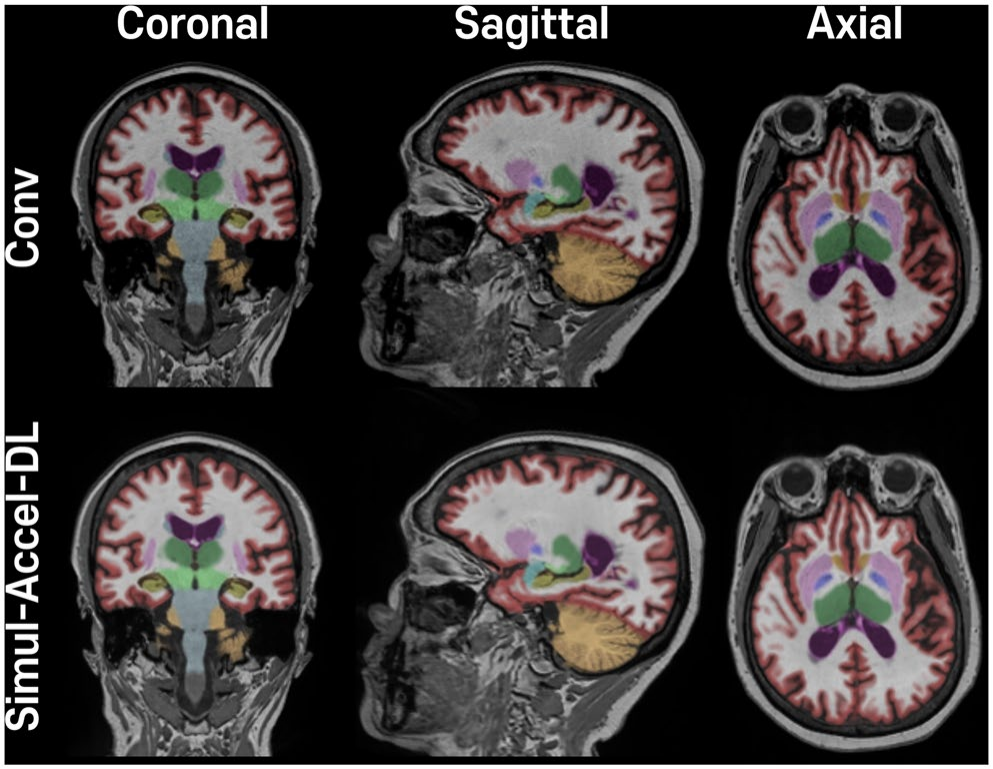
Volumetry results of a 72-year-old man are displayed and processed by NeuroQuant

The simulated acceleration dataset calculated ICC values between Conv and Simul-Accel-DL at three different acceleration levels for each ROI (Table 3). In both NeuroQuant and DeepBrain software, ICC values of the volume measures and normative percentiles were rated excellent (> 0.90), and good (> 0.77), respectively, in Simul-Accel-DL at every acceleration level (Table 3). Additionally, regression coefficients ranged from 0.94 to 1.05, and high R^2^ values (> 0.93) in all ROIs except pallidum analyzed by NeuroQuant revealed the good agreement between Conv and Simul-Accel-DL (Table 4). The representative example of NeuroQuant analysis in Conv and Simul-Accel-DL is shown in Fig. 3, supporting the high ICC values between them. The mean volumes and normative percentile across the subjects are shown good agreement between Conv and Simul-Accel-DL (Supplementary Table S1).

**Table 3:** Intraclass correlation coefficients for volumes and normative percentiles in the simulated acceleration dataset obtained from NeuroQuant and DeepBrain

ROI	Metric	Level 1			Level 2			Level 3		
		ICC	95% CI	*P*-value	ICC	95% CI	*P*-value	ICC	95% CI	*P*-value
*NeuroQuant*										
Total intracranial volume	Volumes	1	0.999-1	< 0.001	1	0.999-1	< 0.001	0.999	0.999-1	< 0.001
	Percentiles	-	-	-	-	-	-	-	-	-
Cortical gray matter	Volumes	0.968	0.945-0.981	< 0.001	0.974	0.952-0.986	< 0.001	0.981	0.964-0.990	< 0.001
	Percentiles	0.909	0.838-0.838	< 0.001	0.915	0.849-0.849	< 0.001	0.925	0.866-0.866	< 0.001
Cerebral white matter	Volumes	0.983	0.970-0.992	< 0.001	0.988	0.978-0.994	< 0.001	0.993	0.987-0.997	< 0.001
	Percentiles	0.789	0.642-0.642	< 0.001	0.807	0.670-0.670	< 0.001	0.844	0.728-0.728	< 0.001
Inferior lateral ventricle	Volumes	0.998	0.997-0.999	< 0.001	0.998	0.997-0.999	< 0.001	0.998	0.997-0.999	< 0.001
	Percentiles	0.992	0.984-0.984	< 0.001	0.990	0.982-0.982	< 0.001	0.988	0.978-0.978	< 0.001
Lateral ventricle	Volumes	1	0.999-1	< 0.001	1	0.999-1	< 0.001	1	0.999-1	< 0.001
	Percentiles	0.999	0.999-1	< 0.001	0.999	0.999-1	< 0.001	0.999	0.999-1	< 0.001
3rd ventricle	Volumes	0.999	0.998-0.999	< 0.001	0.999	0.998-0.999	< 0.001	0.999	0.998-0.999	< 0.001
	Percentiles	0.998	0.996-0.996	< 0.001	0.996	0.993-0.993	< 0.001	0.996	0.993-0.993	< 0.001
4th ventricle	Volumes	0.994	0.989-0.997	< 0.001	0.995	0.991-0.997	< 0.001	0.995	0.992-0.997	< 0.001
	Percentiles	0.987	0.976-0.976	< 0.001	0.991	0.983-0.983	< 0.001	0.991	0.984-0.984	< 0.001
Caudate	Volumes	0.978	0.941-0.995	< 0.001	0.962	0.888-0.996	< 0.001	0.984	0.960-0.995	< 0.001
	Percentiles	0.922	0.860-0.860	< 0.001	0.927	0.869-0.869	< 0.001	0.928	0.872-0.872	< 0.001
Putamen	Volumes	0.985	0.967-0.995	< 0.001	0.981	0.953-0.995	< 0.001	0.987	0.978-0.994	< 0.001
	Percentiles	0.991	0.983-0.983	< 0.001	0.990	0.982-0.982	< 0.001	0.992	0.985-0.985	< 0.001
Pallidum	Volumes	0.912	0.849-0.956	< 0.001	0.913	0.850-0.955	< 0.001	0.905	0.831-0.952	< 0.001
	Percentiles	0.871	0.773-0.773	< 0.001	0.888	0.802-0.802	< 0.001	0.859	0.754-0.754	< 0.001
Thalamus	Volumes	0.983	0.967-0.991	< 0.001	0.988	0.977-0.995	< 0.001	0.986	0.974-0.993	< 0.001
	Percentiles	0.947	0.905-0.905	< 0.001	0.965	0.937-0.937	< 0.001	0.982	0.966-0.966	< 0.001
Amygdala	Volumes	0.984	0.972-0.991	< 0.001	0.982	0.967-0.990	< 0.001	0.981	0.962-0.991	< 0.001
	Percentiles	0.963	0.932-0.932	< 0.001	0.962	0.931-0.931	< 0.001	0.947	0.905-0.905	< 0.001
Hippocampus	Volumes	0.978	0.962-0.988	< 0.001	0.986	0.974-0.992	< 0.001	0.990	0.983-0.995	< 0.001
	Percentiles	0.972	0.949-0.949	< 0.001	0.976	0.957-0.957	< 0.001	0.978	0.959-0.959	< 0.001
*DeepBrain*										
Total intracranial volume	Volumes	1	0.999-1	< 0.001	1	0.999-1	< 0.001	1	0.999-1	< 0.001
	Percentiles	-	-	-	-	-	-	-	-	-
Cortical gray matter	Volumes	0.996	0.993-0.998	< 0.001	0.996	0.992-0.998	< 0.001	0.994	0.990-0.997	< 0.001
	Percentiles	0.990	0.982-0.982	< 0.001	0.995	0.990-0.990	< 0.001	0.993	0.986-0.986	< 0.001
Cerebral white matter	Volumes	0.994	0.989-0.997	< 0.001	0.990	0.982-0.994	< 0.001	0.989	0.981-0.993	< 0.001
	Percentiles	0.976	0.955-0.955	< 0.001	0.954	0.917-0.917	< 0.001	0.949	0.908-0.908	< 0.001
Inferior lateral ventricle	Volumes	1	0.999-1	< 0.001	1	0.999-1	< 0.001	1	0.999-1	< 0.001
	Percentiles	0.999	0.998-1	< 0.001	0.999	0.999-1	< 0.001	0.999	0.999-1	< 0.001
Lateral ventricle	Volumes	1	0.999-1	< 0.001	1	0.999-1	< 0.001	1	0.999-1	< 0.001
	Percentiles	0.999	0.999-1	< 0.001	0.999	0.999-1	< 0.001	0.999	0.999-1	< 0.001
3rd ventricle	Volumes	0.999	0.999-1	< 0.001	1	0.999-1	< 0.001	1	0.999-1	< 0.001
	Percentiles	0.999	0.998-1	< 0.001	0.999	0.998-1	< 0.001	0.999	0.999-1	< 0.001
4th ventricle	Volumes	0.998	0.997-0.999	< 0.001	0.999	0.998-0.999	< 0.001	0.998	0.997-0.999	< 0.001
	Percentiles	0.999	0.997-1	< 0.001	0.999	0.998-1	< 0.001	0.997	0.995-0.995	< 0.001
Caudate	Volumes	0.994	0.987-0.998	< 0.001	0.996	0.990-0.998	< 0.001	0.995	0.987-0.998	< 0.001
	Percentiles	0.990	0.981-0.981	< 0.001	0.990	0.982-0.982	< 0.001	0.987	0.976-0.976	< 0.001
Putamen	Volumes	0.996	0.992-0.999	< 0.001	0.996	0.991-0.999	< 0.001	0.997	0.994-0.998	< 0.001
	Percentiles	0.994	0.989-0.989	< 0.001	0.993	0.987-0.987	< 0.001	0.992	0.985-0.985	< 0.001
Pallidum	Volumes	0.991	0.981-0.995	< 0.001	0.989	0.978-0.995	< 0.001	0.987	0.978-0.993	< 0.001
	Percentiles	0.991	0.984-0.984	< 0.001	0.989	0.980-0.980	< 0.001	0.986	0.974-0.974	< 0.001
Thalamus	Volumes	0.999	0.997-0.999	< 0.001	0.999	0.997-0.999	< 0.001	0.998	0.997-0.999	< 0.001
	Percentiles	0.997	0.995-0.995	< 0.001	0.997	0.995-0.995	< 0.001	0.996	0.992-0.992	< 0.001
Amygdala	Volumes	0.992	0.984-0.997	< 0.001	0.992	0.981-0.997	< 0.001	0.988	0.969-0.997	< 0.001
	Percentiles	0.988	0.979-0.979	< 0.001	0.989	0.979-0.979	< 0.001	0.988	0.978-0.978	< 0.001
Hippocampus	Volumes	0.998	0.996-0.999	< 0.001	0.997	0.995-0.999	< 0.001	0.997	0.994-0.998	< 0.001
	Percentiles	0.994	0.989-0.989	< 0.001	0.993	0.986-0.986	< 0.001	0.989	0.980-0.980	< 0.001

*Note* The intraclass correlation coefficients were estimated between conventional images (Conv) and simulated acceleration images with deep learning-based reconstruction (Simul-Accel-DL) in each region of interest. Acceleration levels 1, 2, and 3 were simulated by 65%, 70%, and 75% undersampling relative to full-sampled acquisition, respectively. ROI = Region of interest, ICC = Intraclass correlation coefficient, and CI = Confidence interval

**Table 4:** Linear regression analysis for volumes in the simulated acceleration dataset obtained from NeuroQuant and DeepBrain

ROI	Level 1		Level 2		Level 3	
	Coefficient	R^2^	Coefficient	R^2^	Coefficient	R^2^
*Volume Analysis by NeuroQuant*						
Total intracranial volume	1.00	1.00	1.00	1.00	1.00	1.00
Cortical gray matter	1.04	0.97	1.02	0.98	0.99	0.99
Cerebral white matter	0.94	0.94	0.95	0.95	0.97	0.96
Inferior lateral ventricle	1.00	1.00	1.00	1.00	0.99	1.00
Lateral ventricle	1.01	1.00	1.01	1.00	1.01	1.00
3rd ventricle	1.00	1.00	1.01	1.00	1.00	1.00
4th ventricle	1.03	0.99	1.05	0.99	1.06	0.99
Caudate	0.98	0.96	1.01	0.93	1.00	0.97
Putamen	0.97	0.97	0.96	0.96	0.95	0.98
Pallidum	1.07	0.85	1.08	0.85	1.05	0.84
Thalamus	0.96	0.97	0.99	0.98	0.98	0.97
Amygdala	1.04	0.97	1.03	0.97	1.02	0.96
Hippocampus	1.03	0.96	1.02	0.97	1.01	0.98
*Volume Analysis by DeepBrain*						
Total intracranial volume	1.00	1.00	1.00	1.00	1.00	1.00
Cortical gray matter	1.00	0.99	1.00	0.99	0.98	0.99
Cerebral white matter	0.97	0.99	0.97	0.98	0.98	0.98
Inferior lateral ventricle	1.00	1.00	1.00	1.00	1.00	1.00
Lateral ventricle	1.00	1.00	1.00	1.00	1.00	1.00
3rd ventricle	1.00	1.00	1.00	1.00	1.00	1.00
4th ventricle	1.02	1.00	1.01	1.00	1.01	1.00
Caudate	1.01	0.99	1.01	0.99	1.00	0.99
Putamen	1.02	0.99	1.02	0.99	1.01	0.99
Pallidum	0.99	0.98	0.99	0.98	1.01	0.97
Thalamus	0.98	1.00	0.98	1.00	0.97	1.00
Amygdala	1.01	0.98	1.02	0.98	1.02	0.98
Hippocampus	1.02	1.00	1.02	1.00	1.02	0.99

*Note* Linear regression coefficient and R-squared values were estimated between conventional images (Conv) and simulated acceleration images with deep learning-based reconstruction (Simul-Accel-DL) in each region of interest. Acceleration levels 1, 2, and 3 were simulated by 65%, 70%, and 75% undersampling relative to full-sampled acquisition, respectively. Conventional images were used as reference to calculate the metrics. ROI = Region of interest

**Fig. 4:**
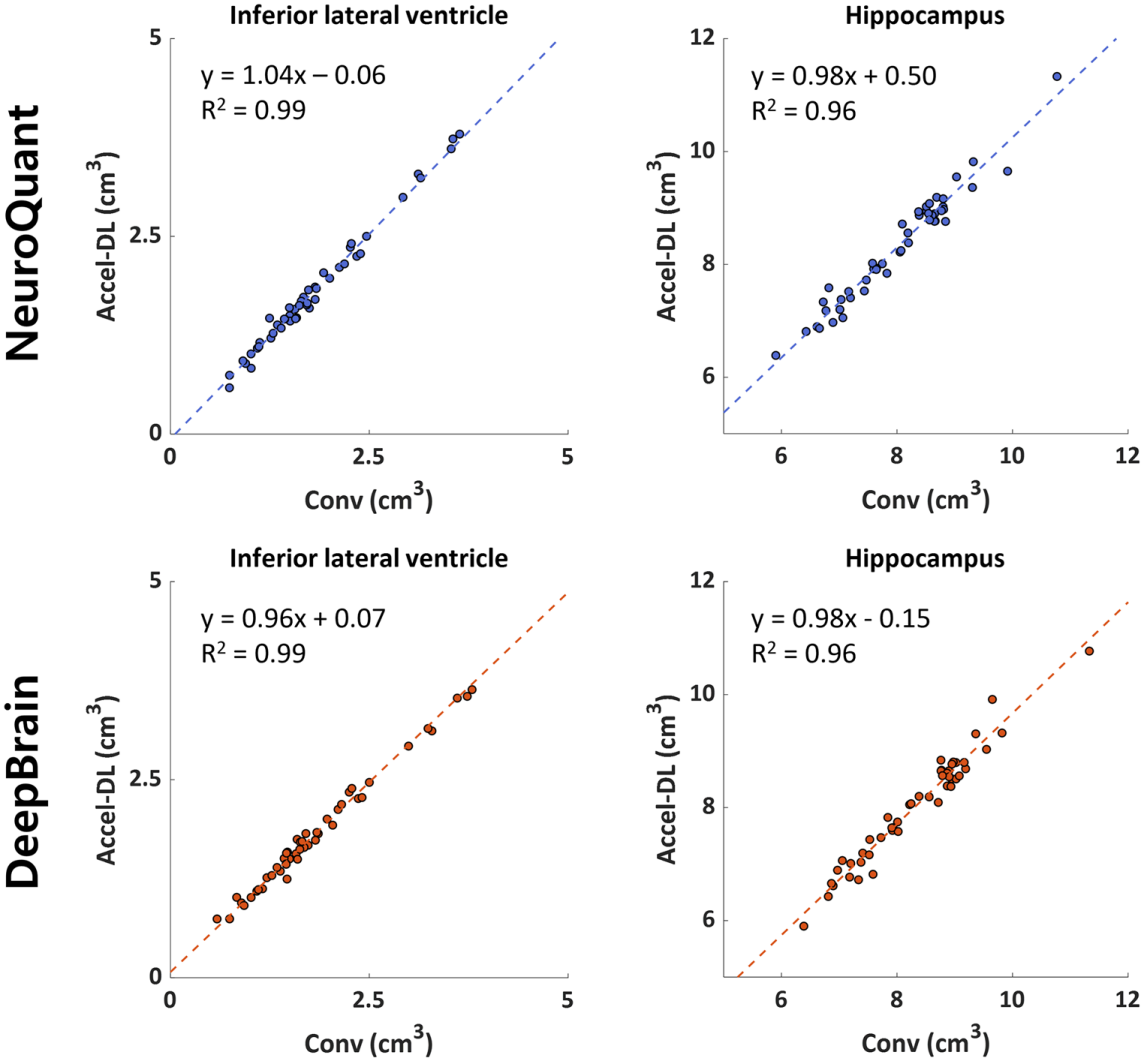
Linear regression analyses were performed between conventional images (Conv) and accelerated scan images with DL-based reconstruction (Accel-DL) in the inferior lateral ventricle and hippocampus. All linear regression lines are close to the line of unity with high R^2^ values, revealing good agreement between the two image reconstructions

**Fig. 5:**
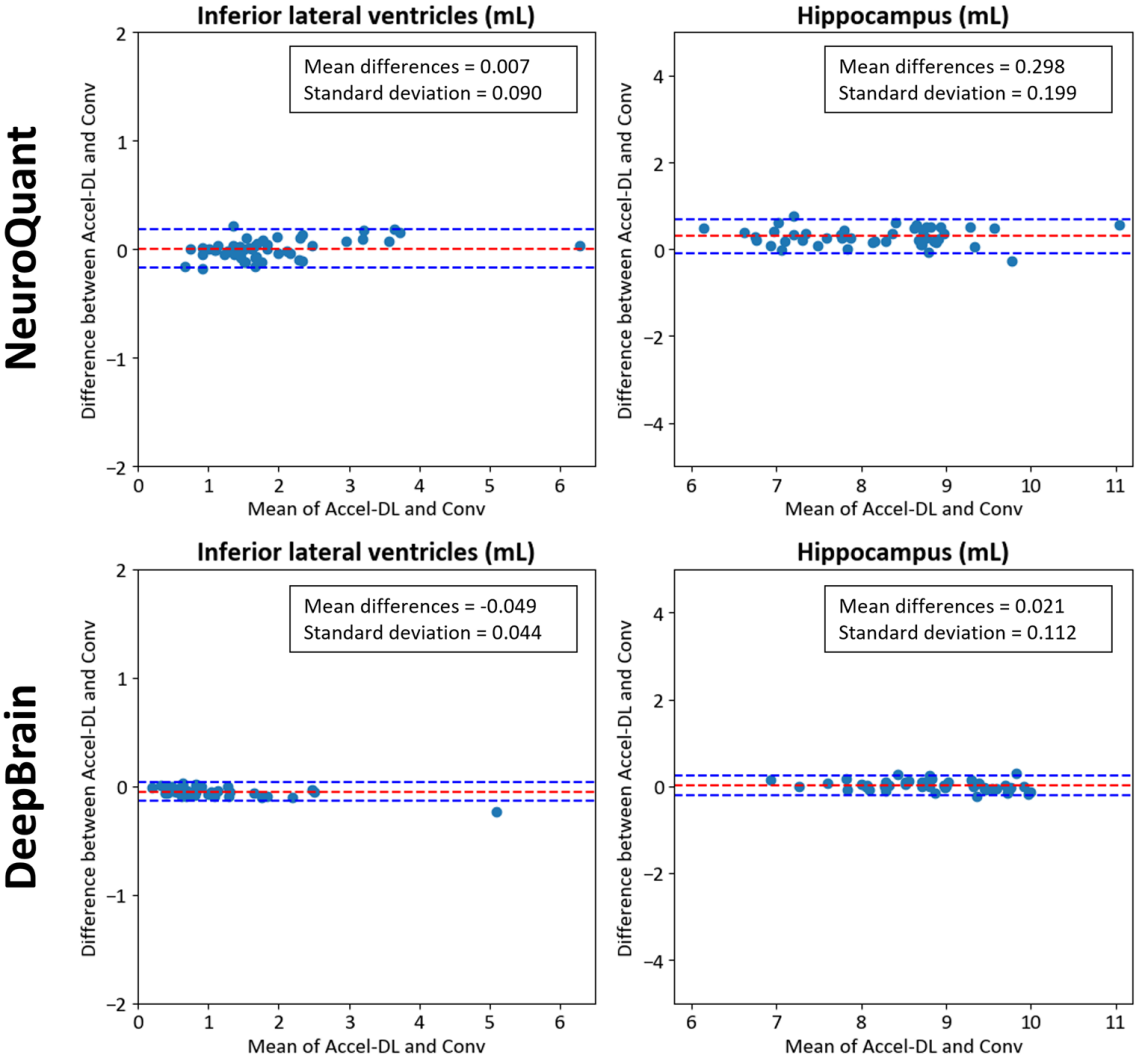
Bland-Altman plots between conventional images (Conv) and accelerated scan images with DL-based reconstruction (Accel-DL) in the inferior lateral ventricle and hippocampus

In the validation dataset, both NeuroQuant and DeepBrain yielded good or excellent inter-scan reliabilities (ICC > 0.87 for volumes; ICC > 0.77 for normative percentiles) in all ROIs (Table 5). The regression coefficients ranged from 0.93 to 1.03, and R^2^ over than 0.92 were estimated in all ROIs except pallidum from both NeuroQuant and DeepBrain (Table 6). In addition, the linear regression (Fig. 4) and Bland-Altman (Fig. 5) analyses for the hippocampus and inferior lateral ventricle demonstrated strong agreement between Conv and Accel-DL for each software (NeuroQuant: mean difference < 0.3 mL, R^2^ > 0.95; DeepBrain: mean differences < 0.02 mL, R^2^ > 0.95). The mean volumes and normative percentile across the subjects were comparable between Conv and Accel-DL, supporting the results from linear regression and Bland-Altman analyses (Supplementary Table S2).

**Table 5:** Intraclass correlation coefficients for volumes and normative percentiles in the validation dataset obtained from NeuroQuant and Deepbrain

ROI	NeuroQuant			DeepBrain		
	ICC	95% CI	*P*-value	ICC	95% CI	*P*-value
*Volumes*						
Total intracranial volume	0.999	0.999-1	< 0.001	0.999	0.998-0.999	< 0.001
Cortical gray matter	0.978	0.964-0.988	< 0.001	0.983	0.972-0.990	< 0.001
Cerebral white matter	0.980	0.963-0.989	< 0.001	0.988	0.981-0.993	< 0.001
Inferior lateral ventricle	0.996	0.989-0.998	< 0.001	0.999	0.996-0.999	< 0.001
Lateral ventricle	1	0.999-1	< 0.001	1	0.999-1	< 0.001
3rd ventricle	0.998	0.995-0.999	< 0.001	0.998	0.997-0.999	< 0.001
4th ventricle	0.988	0.979-0.993	< 0.001	0.995	0.992-0.997	< 0.001
Caudate	0.987	0.980-0.992	< 0.001	0.992	0.984-0.996	< 0.001
Putamen	0.966	0.942-0.979	< 0.001	0.993	0.987-0.997	< 0.001
Pallidum	0.872	0.734-0.948	< 0.001	0.976	0.957-0.987	< 0.001
Thalamus	0.971	0.950-0.982	< 0.001	0.993	0.989-0.996	< 0.001
Amygdala	0.967	0.938-0.983	< 0.001	0.977	0.962-0.987	< 0.001
Hippocampus	0.979	0.966-0.987	< 0.001	0.988	0.980-0.993	< 0.001
*Normative percentiles*						
Cortical gray matter	0.921	0.864-0.864	< 0.001	0.917	0.858-0.858	< 0.001
Cerebral white matter	0.892	0.816-0.816	< 0.001	0.973	0.953-0.953	< 0.001
Inferior lateral ventricle	0.979	0.964-0.964	< 0.001	0.996	0.994-0.994	< 0.001
Lateral ventricle	0.999	0.998-1	< 0.001	0.999	0.999-1	< 0.001
3rd ventricle	0.995	0.991-0.991	< 0.001	0.997	0.995-0.995	< 0.001
4th ventricle	0.977	0.960-0.960	< 0.001	0.992	0.985-0.985	< 0.001
Caudate	0.966	0.941-0.941	< 0.001	0.990	0.982-0.982	< 0.001
Putamen	0.897	0.824-0.824	< 0.001	0.985	0.974-0.974	< 0.001
Pallidum	0.781	0.641-0.641	< 0.001	0.957	0.925-0.925	< 0.001
Thalamus	0.606	0.393-0.393	< 0.001	0.978	0.962-0.962	< 0.001
Amygdala	0.909	0.845-0.845	< 0.001	0.965	0.939-0.939	< 0.001
Hippocampus	0.936	0.890-0.890	< 0.001	0.981	0.967-0.967	< 0.001

*Note* Intraclass correlation coefficient was estimated between conventional images (Conv) and accelerated scan images with deep learning-based reconstruction (Accel-DL) in each region of interest. ROI = Region of interest, ICC = Intraclass correlation coefficient, and CI = Confidence interval

**Table 6:** Linear regression analysis for volumes in the validation dataset obtained from NeuroQuant and DeepBrain

ROI	NeuroQuant		DeepBrain	
	Coefficient	*R* ^2^	Coefficient	*R* ^2^
Total intracranial volume	1.02	1.00	1.01	1.00
Cortical gray matter	0.99	0.96	1.00	0.97
Cerebral white matter	0.98	0.96	0.99	0.98
Inferior lateral ventricle	1.04	0.99	0.96	1.00
Lateral ventricle	1.00	1.00	1.00	1.00
3rd ventricle	1.01	1.00	1.02	1.00
4th ventricle	1.00	0.98	0.97	0.99
Caudate	0.98	0.98	0.97	0.98
Putamen	0.94	0.93	1.01	0.99
Pallidum	0.93	0.76	1.03	0.96
Thalamus	0.96	0.94	1.00	0.99
Amygdala	0.94	0.94	0.94	0.96
Hippocampus	0.98	0.96	0.98	0.96

*Note* Linear regression coefficient and R-squared values were estimated between conventional images (Conv) and accelerated scan images with deep learning-based reconstruction (Accel-DL) in each region of interest. Conventional images were used as reference to calculate the metrics. ROI = Region of interest

## Discussion and conclusion

This study explored the clinical feasibility of accelerated scans with DL-based reconstruction regarding image quality improvement and validation for using volumetric MRI in routine clinical practice. The simulated acceleration dataset was generated by retrospective under-sampling simulation to k-space data to evaluate image quality improvement, followed by DL-based reconstruction. The simulation was performed three times to generate images with 65%, 70%, and 75% acceleration relative to full-sampled acquisition. In all acceleration levels, quantitative error metrics showed that DL-reconstructed images were comparable to (*p* > 0.330) or better than (*p* < 0.001) the images without DL-reconstruction in both SSIM and PSNR. In addition, the improvements in SSIM and PSNR by DL-based reconstruction were increased as the acceleration level increased. When the brain volumetric software was applied to the DL-reconstructed images, the results revealed excellent ICC values in volumes (> 0.90) and good in normative percentiles (> 0.77). In addition, the linear regression analysis in volumes revealed high R^2^ values (> 0.93) at all ROIs except pallidum compared to conventional images at every acceleration level. These results demonstrated the reliability of brain volume measures in accelerated scans with DL-based reconstruction. Moreover, volumetric analysis was performed in the validation dataset consisting of conventional and accelerated scans with DL-based reconstruction. The accelerated scan was acquired with scan times corresponding to simulated acceleration level 3. In volume measures, all ICC and R^2^ values for inter-scan reliability (Conv vs. Accel-DL) were estimated as ICC > 0.9 and R^2^ > 0.93, except the pallidum, rated as ICC = 0.872 and R^2^ = 0.76 in NeuroQuant. Additionally, all ICC values for normative percentiles were estimated as ICC > 0.89 except the pallidum (ICC = 0.781 in NeuroQuant). In Bland-Altman analysis, inferior lateral ventricle and hippocampus revealed good agreement between Conv and Accel-DL (NeuroQuant: mean differences < 0.30, standard deviation < 0.20; DeepBrain: mean differences < 0.05, standard deviation < 0.12).

In this research, we have demonstrated that substantial acceleration in image acquisition, by up to 75% compared to full-sampled scans, does not compromise the volumetric results derived from MP-RAGE scans. The standard full-sampled MP-RAGE scan typically requires approximately 5 min to complete. By employing acceleration protocols, this duration can be significantly reduced without degradation of the data quality for brain volumetric analysis. This finding has considerable implications for routine 3D MP-RAGE brain scans, where scan time reduction can notably decrease the likelihood of motion artifacts, which often compromise the scan quality. Additionally, shorter scan durations could significantly enhance patient comfort during the procedure, which may improve cooperation and overall patient experience. This study, therefore, indicates a promising path for implementing accelerated MP-RAGE protocols in routine clinical practice.

The hippocampus and inferior lateral ventricles are important in brain volume measurements. Their significance lies in that these volumes form the basis for estimating quantitative metrics such as hippocampal occupancy and medial temporal lobe atrophy score, supporting AD diagnosis. Therefore, we assessed the inter-scan reliability and linear regression analysis for these volumes to compare Conv and Accel-DL. Using two volumetric software, our results showed excellent inter-scan reliability between Conv and Accel-DL for the regions. As a result, accelerated scans with DL-based reconstruction present a promising tool for highly reliable measurements of brain atrophy irrespective of the type of software, particularly in the hippocampus and inferior lateral ventricle. These advancements potentially improve our capacity to detect AD and related neurodegenerative disorders during their prodromal stages [16, 28].

Our estimation of ICC values for the pallidum between Conv and Accel-DL images resulted in 0.872 for volumes and 0.781 for normative percentiles at NeuroQuant analysis, indicating low inter-scan reliability compared to other brain regions (Other ICCs > 0.96 for volumes, > 0.89 in normative percentiles). Additionally, estimated R^2^ values at pallidum (= 0.76) in NeuroQuant is lower than other ROIs (R^2^ values > 0.92). Such findings align with previous studies comparing various volumetric algorithms [29, 30]. The primary culprit behind this lower inter-scan reliability is the similar contrast observed between the pallidum and white matter in T1-weighted MRI scans. This introduces significant challenges to achieving precise and consistent volumetric measurements. Given these observations, future research needs to be conducted to explore further sequence optimization or improvements to the volumetric algorithms, aiming to address the reliability issues of pallidum.

Comparing NeuroQuant and DeepBrain analyses, the DeepBrain results have shown comparable or better error metrics from both Simul-Accel-DL and Accel-DL. Particularly, DeepBrain showed higher R^2^ values at pallidum between Conv and Accel-DL compared to NeuroQuant (0.96 vs. 0.76). This is mainly because each software performs brain volumetry based on different algorithms. NeuroQuant utilizes the per-defined atlas to assign each voxel to the corresponding anatomic label based on probabilistic calculation [16], while DeepBrain utilizes a deep learning-based segmentation model [17]. In addition, DeepBrain reveals high ICC values in normative percentiles between Conv and Accel-DL compared to NeuroQuant (0.957 vs. 0.781). Each software constructs a normative database from different racial populations, leading to variation in estimated normative percentiles. This variation has been reported in the previous studies [30, 31].

There are a few limitations to our study. First, the study was conducted within a single center, using a single vendor system, which may limit the generalizability of the results to other settings or equipment. Further, we restricted our evaluation to two clinically available volumetric software, NeuroQuant and DeepBrain. Despite their wide usage, these two do not encompass all possible volumetric analysis options, and the findings could differ with other software. Furthermore, the sample size of our study was relatively small to cover the diversity of the patient population or brain morphology. Hence, further large-scale, multi-center studies employing a diverse range of volumetric software need to be performed to consolidate our findings.

In conclusion, our study demonstrated the reliability of brain volumetric results in accelerated MRI scans with DL-based reconstruction. The DL reconstruction model performed accelerated scanned MR images by up to 75% relative to the full-sampled acquisition, and the reconstructed images showed reliability in volumetric analysis with two clinically available software applications. This finding supports the clinical feasibility of accelerated MRI scans with DL-based reconstruction for using volumetric quantitative MRI in routine clinical practice.

## Electronic supplementary material

Below is the link to the electronic supplementary material.

Supplementary Material 1

## Data Availability

The data that support the findings of this study are available from the corresponding author upon reasonable request.
