# Supplementary material for: Reliability of brain volume measures of accelerated 3D T1-weighted images with deep learning-based reconstruction
Source: Neuroradiology. 2024 Sep 24;67(1):171–82. doi: 10.1007/s00234-024-03461-5 (PMC11802604; doi:10.1007/s00234-024-03461-5)
Supplement: Supplementary file 1 — Supplementary Material 1 [file 234_2024_3461_MOESM1_ESM.docx]

**Supplementary Table S1.** Comparison of volumes and normative percentiles in the simulated acceleration dataset obtained from NeuroQuant and DeepBrain

| Volume Analysis by NeuroQuant | | | | | | | | | |  | | |  |
| --- | --- | --- | --- | --- | --- | --- | --- | --- | --- | --- | --- | --- | --- |
| ROI | Volumes (mL) | | | | Normative Percentile | | | | | | | |  |
|  | Conv | Level1 | Level2 | Level3 | Conv | Level1 | | Level2 | | | Level3 | |  |
| Total intracranial volume | 1548.9 ± 151.5 | 1549.7 ± 150.9 | 1550.6 ± 151.0 | 1552.5 ± 152.1 | - | - | | - | | | - | |  |
| Cortical GM | 454.87 ± 55.48 | 465.24 ± 58.65 | 467.08 ± 57.46 | 470.29 ± 55.48 | 24.2 ± 21.6 | 33.9 ± 27.5 | | 34.8 ± 27.2 | | | 36.7 ± 25.9 | |  |
| Cerebral WM | 500.63 ± 53.14 | 485.01 ± 51.48 | 483.99 ± 51.75 | 483.30 ± 52.41 | 88.1 ± 15.9 | 78.8 ± 23.7 | | 78.0 ± 23.4 | | | 77.6 ± 22.7 | |  |
| Inferior lateral ventricle | 2.50 ± 1.29 | 2.54 ± 1.28 | 2.57 ± 1.29 | 2.57 ± 1.27 | 64.2 ± 31.4 | 66.6 ± 29.9 | | 67.4 ± 29.4 | | | 67.3 ± 29.9 | |  |
| Lateral ventricle | 36.83 ± 17.98 | 36.58 ± 18.09 | 36.59 ± 18.16 | 36.64 ± 18.16 | 54.2 ± 27.6 | 53.4 ± 28.0 | | 53.5 ± 28.0 | | | 53.5 ± 28.0 | |  |
| 3^rd^ ventricle | 2.06 ± 0.71 | 2.06 ± 0.72 | 2.06 ± 0.72 | 2.07 ± 0.72 | 58.1 ± 24.9 | 58.2 ± 24.9 | | 58.5 ± 25.0 | | | 58.5 ± 25.1 | |  |
| 4th ventricle | 1.98 ± 0.55 | 1.98 ± 0.57 | 1.98 ± 0.58 | 1.96 ± 0.58 | 53.5 ± 27.0 | 52.8 ± 28.1 | | 52.5 ± 28.4 | | | 51.1 ± 28.5 | |  |
| Caudate | 5.77 ± 1.13 | 5.52 ± 1.14 | 5.52 ± 1.19 | 5.54 ± 1.15 | 59.7 ± 31.1 | 49.9 ± 33.5 | | 50.2 ± 33.7 | | | 49.8 ± 34.0 | |  |
| Putamen | 11.87 ± 1.46 | 11.78 ± 1.43 | 11.78 ± 1.43 | 11.86 ± 1.40 | 86.8 ± 18.4 | 86.0 ± 18.6 | | 86.2 ± 18.2 | | | 86.9 ± 17.5 | |  |
| Pallidum | 0.86 ± 0.25 | 0.91 ± 0.29 | 0.91 ± 0.29 | 0.92 ± 0.29 | 6.6 ± 8.8 | 8.7 ± 11.1 | | 8.3 ± 10.9 | | | 9.1 ± 11.2 | |  |
| Thalamus | 16.41 ± 1.77 | 16.15 ± 1.73 | 16.22 ± 1.77 | 16.16 ± 1.76 | 96.1 ± 7.0 | 95.0 ± 9.4 | | 95.2 ± 8.7 | | | 95.5 ± 7.7 | |  |
| Amygdala | 3.14 ± 0.42 | 3.20 ± 0.44 | 3.20 ± 0.43 | 3.21 ± 0.43 | 64.6 ± 27.8 | 69.1 ± 26.5 | | 68.5 ± 26.3 | | | 70.0 ± 25.0 | |  |
| Hippocampus | 7.47 ± 1.02 | 7.51 ± 1.08 | 7.51 ± 1.06 | 7.58 ± 1.04 | 73.3 ± 26.3 | 74.0 ± 25.7 | | 74.2 ± 26.0 | | | 76.0 ± 25.2 | |  |
|  |  |  |  |  |  |  | |  | | |  | |  |
| Volume Analysis by DeepBrain | | | | | | | | | |  | | |  |
| ROI | Volumes (mL) | | | | Normative Percentile | | | | | | | | |
|  | Conv | Level1 | Level2 | Level3 | Conv | | Level1 | | Level2 | | | Level3 | |
| Total intracranial volume | 1493.5 ± 142.9 | 1494.3 ± 142.7 | 1494.3 ± 142.8 | 1493.6 ± 142.8 | - | | - | | - | | | - | |
| Cortical GM | 439.38 ± 46.38 | 441.28 ± 46.39 | 441.82 ± 46.37 | 441.76 ± 45.77 | 88.6 ± 24.2 | | 89.5 ± 24.0 | | 89.5 ± 23.8 | | | 89.6 ± 23.6 | |
| Cerebral WM | 451.39 ± 46.30 | 452.13 ± 45.26 | 451.93 ± 45.34 | 453.13 ± 45.86 | 53.7 ± 34.0 | | 53.1 ± 32.9 | | 52.9 ± 32.9 | | | 54.6 ± 32.2 | |
| Inferior lateral  ventricle | 1.34 ± 0.92 | 1.34 ± 0.92 | 1.34 ± 0.91 | 1.33 ± 0.91 | 46.8 ± 25.4 | | 46.5 ± 25.4 | | 46.6 ± 25.5 | | | 46.4 ± 25.4 | |
| Lateral ventricle | 32.10 ± 16.81 | 32.06 ± 16.83 | 32.09 ± 16.84 | 32.16 ± 16.82 | 49.0 ± 29.7 | | 48.8 ± 29.7 | | 48.9 ± 29.7 | | | 49.2 ± 29.7 | |
| 3^rd^ ventricle | 3.24 ± 0.86 | 3.23 ± 0.85 | 3.24 ± 0.85 | 3.25 ± 0.86 | 64.7 ± 31.4 | | 64.1 ± 31.3 | | 64.3 ± 31.3 | | | 64.8 ± 31.4 | |
| 4th ventricle | 1.61 ± 0.52 | 1.63 ± 0.53 | 1.64 ± 0.53 | 1.65 ± 0.53 | 32.0 ± 30.5 | | 33.1 ± 30.7 | | 33.2 ± 30.7 | | | 33.9 ± 30.5 | |
| Caudate | 6.96 ± 0.99 | 6.86 ± 1.01 | 6.87 ± 1.00 | 6.86 ± 0.99 | 48.5 ± 36.0 | | 45.1 ± 36.5 | | 45.1 ± 36.5 | | | 45.0 ± 36.4 | |
| Putamen | 9.76 ± 1.18 | 9.65 ± 1.20 | 9.65 ± 1.20 | 9.64 ± 1.20 | 46.3 ± 31.0 | | 42.7 ± 31.0 | | 42.5 ± 31.1 | | | 42.4 ± 31.2 | |
| Pallidum | 2.74 ± 0.28 | 2.72 ± 0.28 | 2.72 ± 0.28 | 2.73 ± 0.28 | 64.2 ± 32.9 | | 61.6 ± 34.6 | | 61.1 ± 34.9 | | | 62.5 ± 34.5 | |
| Thalamus | 14.61 ± 1.44 | 14.59 ± 1.42 | 14.60 ± 1.42 | 14.64 ± 1.40 | 76.9 ± 27.8 | | 76.0 ± 28.1 | | 76.2 ± 28.1 | | | 77.8 ± 27.1 | |
| Amygdala | 3.11 ± 0.31 | 3.10 ± 0.32 | 3.10 ± 0.32 | 3.11 ± 0.33 | 35.0 ± 28.8 | | 33.9 ± 29.9 | | 34.4 ± 30.0 | | | 35.5 ± 29.8 | |
| Hippocampus | 8.51 ± 0.83 | 8.47 ± 0.85 | 8.48 ± 0.85 | 8.44 ± 0.84 | 48.7 ± 30.4 | | 46.8 ± 30.9 | | 46.9 ± 30.5 | | | 45.2 ± 30.3 | |

Note: Volume measures and normative percentiles were estimated at conventional images (Conv) and simulated acceleration images with deep learning-based reconstruction (Simul-Accel-DL) in each region of interest. Acceleration levels 1, 2, and 3 were simulated by 65%, 70%, and 75% undersampling relative to full-sampled acquisition, respectively. ROI = Region of interest, GM = Gray matter, WM = White matter.

**Supplementary Table S2.** Comparison of volumes and normative percentiles in the validation dataset obtained from NeuroQuant and DeepBrain

| Volume Analysis by NeuroQuant | | | | | | | |  |  |  |
| --- | --- | --- | --- | --- | --- | --- | --- | --- | --- | --- |
| ROI | Volume measurements (mL) | | | | Normative percentiles | | | |  |  |
|  | Conv | | Accel-DL | | Conv | | Accel-DL | |  |  |
| Total intracranial volume | 1561.13 ± 113.54 | | 1579.04 ± 115.40 | | - | | - | |  |  |
| Cortical GM | 491.90 ± 42.69 | | 508.29 ± 43.00 | | 38.4 ± 23.2 | | 49.2 ± 25.7 | |  |  |
| Cerebral WM | 492.53 ± 44.74 | | 486.05 ± 45.05 | | 82.6 ± 17.8 | | 73.2 ± 23.2 | |  |  |
| Inferior lateral ventricle | 1.89 ± 0.96 | | 1.90 ± 1.00 | | 58.6 ± 27.0 | | 57.4 ± 27.0 | |  |  |
| Lateral ventricle | 28.83 ± 14.36 | | 28.62 ± 14.37 | | 45.5 ± 27.6 | | 43.7 ± 27.9 | |  |  |
| 3^rd^ ventricle | 1.68 ± 0.62 | | 1.67 ± 0.63 | | 45.5 ± 27.4 | | 43.6 ± 27.6 | |  |  |
| 4th ventricle | 1.94 ± 0.51 | | 1.92 ± 0.52 | | 56.2 ± 25.1 | | 53.2 ± 26.0 | |  |  |
| Caudate | 6.00 ± 1.07 | | 5.86 ± 1.06 | | 66.8 ± 27.7 | | 59.0 ± 30.3 | |  |  |
| Putamen | 12.56 ± 1.15 | | 12.70 ± 1.11 | | 95.1 ± 6.2 | | 95.3 ± 5.1 | |  |  |
| Pallidum | 1.25 ± 0.28 | | 1.25 ± 0.30 | | 18.7 ± 16.2 | | 18.8 ± 16.3 | |  |  |
| Thalamus | 16.80 ± 2.00 | | 16.38 ± 1.97 | | 97.5 ± 3.4 | | 94.5 ± 7.7 | |  |  |
| Amygdala | 3.34 ± 0.51 | | 3.44 ± 0.49 | | 70.5 ± 26.7 | | 77.0 ± 25.9 | |  |  |
| Hippocampus | 8.02 ± 0.97 | | 8.32 ± 0.96 | | 79.3 ± 22.5 | | 85.2 ± 17.5 | |  |  |
|  |  | |  | |  | |  | |  |  |
| Volume Analysis by DeepBrain | | | | | | | |  |  |  |
| ROI | Volume measurements (mL) | | | Normative percentiles | | | | |  |  |
|  | Conv | Accel-DL | | Conv | | Accel-DL | | |  |  |
| Total intracranial volume | 1498.90 ± 107.71 | 1512.78 ± 109.30 | | - | | - | | |  |  |
| Cortical GM | 450.88 ± 31.82 | 459.17 ± 32.24 | | 85.3 ± 21.2 | | 88.0 ± 19.5 | | |  |  |
| Cerebral WM | 473.44 ± 42.29 | 477.33 ± 42.39 | | 60.7 ± 34.9 | | 59.6 ± 34.0 | | |  |  |
| Inferior lateral  ventricle | 1.01 ± 0.81 | 0.96 ± 0.78 | | 50.6 ± 25.8 | | 47.9 ± 25.5 | | |  |  |
| Lateral ventricle | 22.30 ± 12.81 | 22.30 ± 12.85 | | 47.7 ± 28.3 | | 47.1 ± 28.4 | | |  |  |
| 3^rd^ ventricle | 1.49 ± 0.53 | 1.51 ± 0.55 | | 59.6 ± 31.1 | | 60.3 ± 31.0 | | |  |  |
| 4th ventricle | 1.76 ± 0.50 | 1.74 ± 0.49 | | 50.6 ± 30.8 | | 47.9 ± 30.6 | | |  |  |
| Caudate | 7.22 ± 0.81 | 7.21 ± 0.79 | | 51.0 ± 33.4 | | 47.3 ± 33.6 | | |  |  |
| Putamen | 10.57 ± 1.09 | 10.54 ± 1.11 | | 55.7 ± 30.6 | | 50.6 ± 31.5 | | |  |  |
| Pallidum | 2.86 ± 0.32 | 2.87 ± 0.34 | | 65.6 ± 27.9 | | 62.9 ± 29.6 | | |  |  |
| Thalamus | 14.77 ± 1.28 | 15.02 ± 1.30 | | 50.8 ± 32.9 | | 55.7 ± 32.9 | | |  |  |
| Amygdala | 3.31 ± 0.33 | 3.30 ± 0.31 | | 38.5 ± 30.9 | | 32.9 ± 29.2 | | |  |  |
| Hippocampus | 8.85 ± 0.75 | 8.87 ± 0.71 | | 29.3 ± 27.9 | | 26.9 ± 26.5 | | |  |  |

Note: Volume measurements and normative percentiles were estimated at conventional images (Conv) and accelerated scan images with deep learning-based reconstruction (Accel-DL) in each region of interest. ROI = Region of interest, GM = Gray matter, WM = White matter.
